# MOC-Diatomite Composites Filled with Multi-Walled Carbon Nanotubes

**DOI:** 10.3390/ma14164576

**Published:** 2021-08-15

**Authors:** Milena Pavlíková, Martina Záleská, Adam Pivák, Ondřej Jankovský, Anna-Marie Lauermannová, Michal Lojka, Filip Antončík, Zbyšek Pavlík

**Affiliations:** 1 Department of Materials Engineering and Chemistry, Faculty of Civil Engineering, Czech Technical University in Prague, Thákurova 7, 166 29 Prague 6, Czech Republic; martina.zaleska@fsv.cvut.cz (M.Z.); adam.pivak@fsv.cvut.cz (A.P.); pavlikz@fsv.cvut.cz (Z.P.); 2 Department of Inorganic Chemistry, Faculty of Chemical Technology, University of Chemistry and Technology, Technická 5, 166 28 Prague 6, Czech Republic; ondrej.jankovsky@vscht.cz (O.J.); Anna-marie.Lauermannova@vscht.cz (A.-M.L.); michal.lojka@vscht.cz (M.L.); Filip.Antoncik@vscht.cz (F.A.)

**Keywords:** magnesium oxychloride cement, multi-walled carbon nanotubes, diatomite, structure analysis, mechanical and thermal performance, thermal stability

## Abstract

The studies focusing on magnesium oxychloride cement (MOC) composites have recently become fairly widespread because of MOC’s excellent mechanical properties and environmental sustainability. Numerous fillers, admixtures and nano-dopants were studied in order to improve the overall performance of MOC-based derivatives. Some of them exhibited specific flaws, such as a tendency to aggregate, increase in porosity, aeration of the composite matrix, depreciation in water resistance and mechanical strength, etc. In this manuscript, MOC-based composites doped by multi-walled carbon nanotubes (MWCNTs) are designed and tested. In order to modify the final properties of composites, diatomite was admixed as partial substitution of MgO, which was used in the composition of the researched material in excess, i.e., the majority of MgO constituted part of MOC and the rest served as fine filler. The composites were subjected to the broad experimental campaign that covered SEM (scanning electron microscopy), EDS (energy dispersive spectroscopy), HR-TEM (high-resolution transmission electron microscopy), XRD (X-ray diffraction), OM (optical microscopy) and STA-MS (simultaneous thermal analysis with mass spectroscopy). For 28 days hardened samples, macrostructural and microstructural parameters, mechanical properties, hygric and thermal characteristics were experimentally assessed. The incorporation of MWCNTs and diatomite resulted in the significant enhancement of composites’ compactness, mechanical strength and stiffness and reduction in water absorption and rate of water imbibition. The thermal properties of the enriched MOC composites yielded interesting values and provided information for future modification of thermal performance of MOC composites with respect to their specific use in practice, e.g., in passive moderation of indoor climate. The combination of MWCNTs and diatomite represents a valuable modification of the MOC matrix and can be further exploited in the design and development of advanced building materials and components.

## 1. Introduction

In recent decades, the major incentive for the uptake and development of MOC (magnesium oxychloride cement) composites has been motivated from an environmental standpoint. In comparison to PC production, lower calcination temperature are required for MgO production; thus, energy savings associated with these reduced temperatures have led investigators to imagine MgO-based cement as being an eco-friendly binder in the future. Moreover, the ability of MgO to react with atmospheric CO_2_ creating carbonates or hydroxycarbonates satisfies the image of so-called “carbon-neutral” cement, which is able to absorb nearly the same amount of CO_2_ during its service life as was emitted during its manufacturing. Commercially produced MgO, as one of the raw materials important for the MOC synthesis, is not mined directly but is generally obtained using a dry route, which means the calcination of mined magnesite deposits (MgCO_3_). Moreover, a wet route can be applied, in the form of the precipitations from magnesium-bearing brine or seawater, but it is more energy-consuming [1]. Bilinski et al. observed [2] that the setting time and strength of MOC can vary as a function of calcination conditions, which also strongly affects the reactivity of MgO; thus, it is the final phase formation in the MOC composite structure [3,4]. The ability of MOC cements to bind and incorporate large quantities of diverse filler materials ranging from granite to dust, with organic or inorganic origins and sizes from nanometer to centimeter, allows the creation of MOC composite materials with required properties [5,6,7]. However, the popularity of MOC has sharply declined because of its poor water resistance and subsequent degradation during service life [8]. The reason is that binding phases easily react with water molecules forming more voluminous hydroxides resulting in the loss of the strength of MOC in moist conditions. For that reason, it is important to create material with a defined porous structure and low open porosity. This can be achieved by the incorporation of microfillers into a composite mixture. As we confirmed in our previous works, the addition of fine particles results in structures with low porosity with improved mechanical strength, reduced water ingress and increased resistance against water damage [9,10,11]. The positive effect of microfiller addition can be further strengthened by the application of reactive particles, which can form stable hydrated products. For that reason, it can be utilized. One example is diatomite containing amorphous SiO_2_, and it is able to create M-S-H phases adequate to C-S-H phases in PC [12]. Moreover, the incorporation of graphene nanoplatelets enables the production of materials with the decreased porosity and greatly improved mechanical resistance.

In this paper, we encapsulated our experience to develop novel ecofriendly MOC composite material with low porosity, reduced water ingress and enhanced mechanical properties. In order to decrease porosity and improve mechanical resistance, carbon nanotubes were incorporated in the amount of 0.2 wt.% of the whole composite mixture. In order to explore the most effective dosage of microfiller, we doped MOC with diatomite in the amount of 10, 20 and 30 wt.% of the excess mass of MgO, which served originally as fine filler. The raw materials and developed composites were characterized using a variety of methods, including X-ray powder diffraction (XRD), high-resolution transmission electron microscopy (HR-TEM), scanning electron microscopy (SEM), energy dispersive spectroscopy (EDS) and Fourier transform-infrared spectroscopy (FT-IR). The hardened composites concerned their thermal, structural, mechanical and hygric properties tested.

## 2. Materials and Methods

The materials used in this work for binder preparation are presented in the Supplementary Materials.

The mixture properties of the prepared samples are summarized in Table 1. Let us note that MgO was added in excess; its unreacted part served as a filler. MOC-REF is a reference mixture without CNT (carbon nanotubes) and diatomite. In all samples marked CNT, the carbon nanotubes were used in the amount of 0.2 wt.% of the whole mixture. Samples termed MOC-CNT-D10, MOC-CNT-D20 and MOC-CNT-D30 contained diatomite as partial replacement of the over stoichiometric MgO in the amount of 10 wt.%, 20wt.% and 30 wt.%, respectively.

MgCl_2_·6H_2_O was dissolved in tap water. This solution was used for the dispersion of MWCNTs using Ultra Turrax T-18 (IKA, Staufen, Germany); the process took 5 min at 10,000 rpm. The obtained suspension/plain solution of MgCl_2_ was mixed with MgO/MgO-diatomite accordingly to the mixture prescription. The total mixing time in the planetary type mortar mixer (ELE) was 5 min. The fresh mixtures were then cast into steel molds with dimensions of 40 mm × 40 mm × 160 mm, unmolded after 24 h and cured in the air atmosphere at laboratory conditions (temperature of (23 ± 2) °C and relative humidity of (50 ± 5) %) for the next 27 days.

Diatomite and MgO used in this work were characterized by specific density, powder density, Blaine fineness and particle size distribution.

The prepared samples were analyzed by multiple analytical methods in order to determine their chemical, structural, mechanical, hygric and thermal properties. In order to analyze the fracture surface of the samples, optical microscopy (OM) was used. The phase composition was determined by using X-ray diffraction (XRD). For the study of the surface morphology, scanning electron microscopy (SEM) was used. In order to reach even higher magnification the high-resolution transmission, electron microscopy (HR-TEM) was used. The elemental compositions of the samples as well as the elemental maps were obtained by using energy dispersive spectroscopy (EDS). In order to study the mechanism of formation of the various composites based on the dosage of diatomite, the Fourier-transform infrared spectroscopy was used. The thermal behavior of the samples up to 800 °C was analyzed by using simultaneous thermal analysis in combination with mass spectrometry. The macrostructural and microstructural parameters were determined using helium pycnometry and mercury porosimetry. All the samples also underwent a series of mechanical tests to determine their flexural strength *f_f_* (MPa), compressive strength *f_c_* (MPa) and the dynamic modulus of elasticity *E_d_* (GPa). Moreover, the water transport parameters, which are crucial for the MOC-based composites, were studied. Finally, the thermal conductivity and the volumetric heat capacity were measured using a transient plane source technique. More detailed information about the analytical methods is provided in the Supplementary Materials. 

## 3. Results

The fundamental physical characteristics of diatomite and MgO are given in Table 2. The specific and powder densities of diatomite were much lower compared to those of MgO. This provides evidence of the high porosity of the fossilized diatomite frustules. As MgO was found to be much finer than diatomite, its large specific surface can be attributed to its microporous nature.

**Figure 1:**
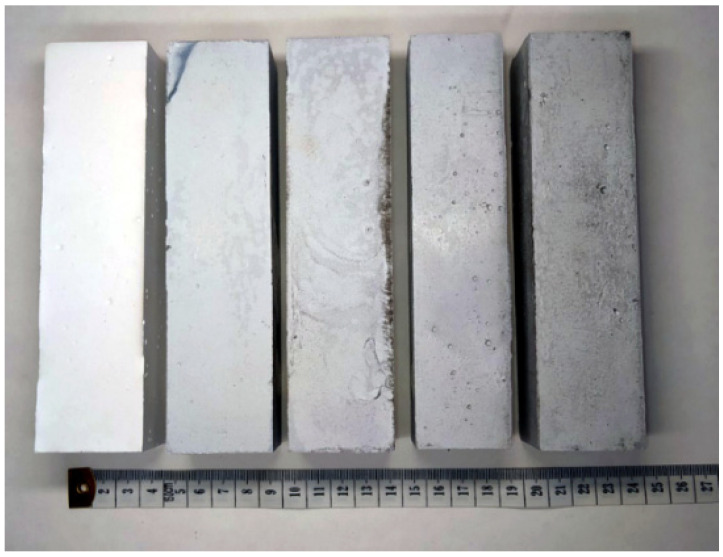
The photograph of the prepared prismatic specimens; from left MOC-REF, MOC-REF-CNT-0.2, MOC-CNT-10, MOC-CNT-D20 and MOC-CNT-D30.

The photo of the hardened prismatic specimens is shown in Figure 1. The use of CNTs and diatomite in composite mixtures did not negatively affect the original appearance of the cast prisms.

**Figure 2:**
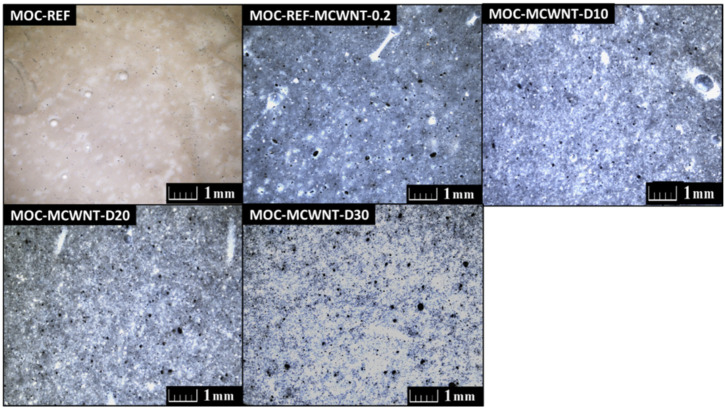
The photograph of the prepared composites obtained from OM.

The fracture surface of the obtained samples was analyzed by using optical microscopy in order to detect the compactness and possible defects in the structure of the composites. The samples present as compact, with no visible defects and with homogenously dispersed additives. In all the samples, there is visible discoloration which is caused by the use of the MWCNTs. The obtained photographs are presented in Figure 2.

**Figure 3:**
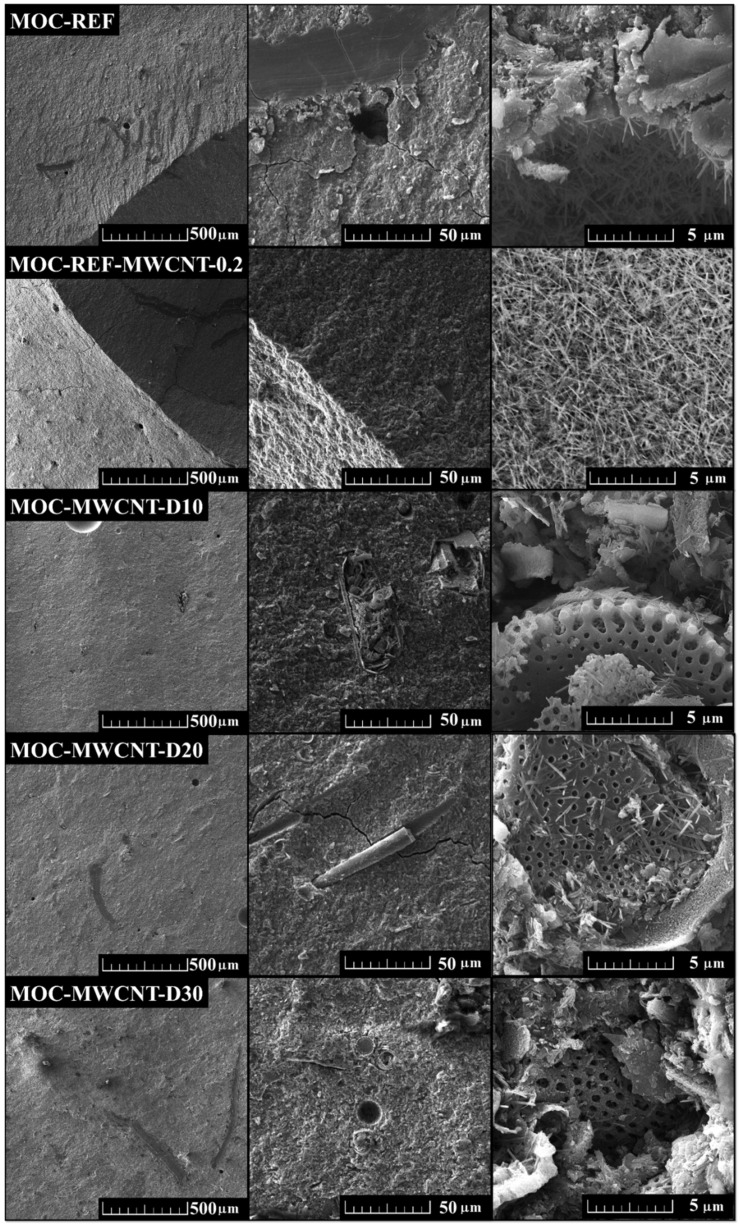
The SEM micrographs of the obtained MOC-based composite materials.

The SEM analysis was used to study the microstructure and morphology of the prepared composites. The micrographs with smaller magnification show the compact structure of the composites with some air bubbles. At higher magnifications, there are visible needle-shaped crystals which are typical of the MOC-type materials. These form in the bubbles and on the diatomite particles, which act as nucleation centers. The obtained micrographs are shown in Figure 3.

**Figure 4:**
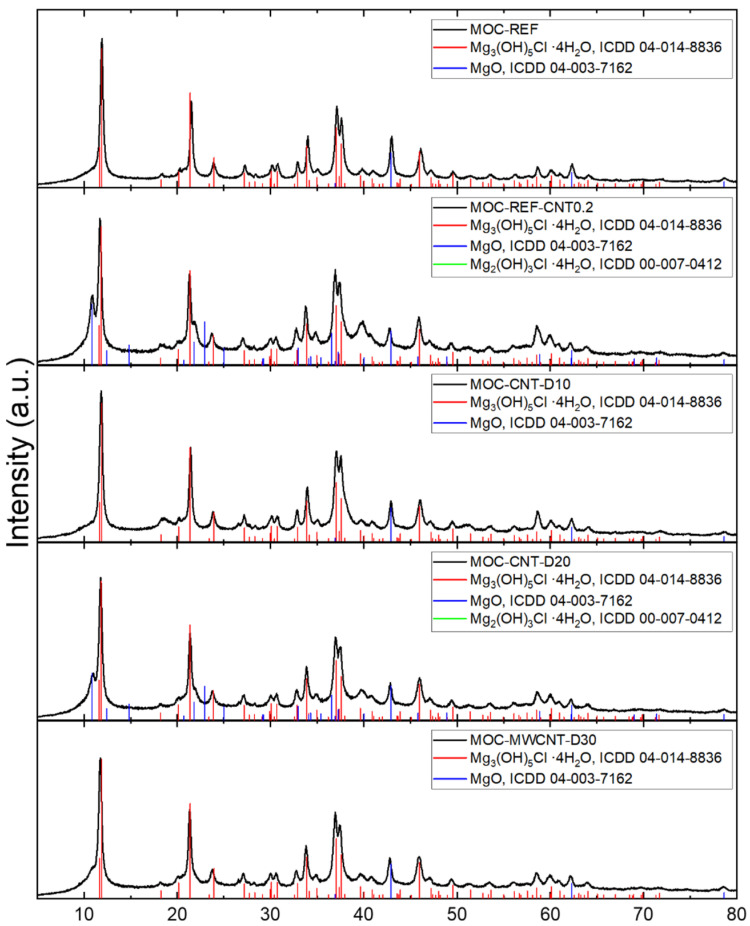
The diffractograms of the samples MOC-REF, MOC-REF-CNT0.2, MOC-CNT-D10, MOC-CNT-D20 and MOC-CNT-30.

The phase composition of the samples was studied by X-ray diffraction. All diffractograms have shown the content of the MOC phase 5 (Mg_3_(OH)_5_Cl∙4H_2_O, ICDD 04-014-8836) and MgO (ICDD 04-003-7162), which were used in excess as a part of the filler. The samples MOC-REF-CNT0.2 and MOC-CNT-D10 also showed that they also contain MOC phase 3 (Mg_2_(OH)_3_Cl∙4H_2_O, ICDD 00-007-0412). Its formation was caused by the local lack of MgCl_2_ due to its aggregation with the carbon nanotubes. The carbon nanotubes are not present in the diffractograms because of their low content and the diatomite is not visible due to its amorphous character. The diffraction patterns are shown in Figure 4.

**Figure 5:**
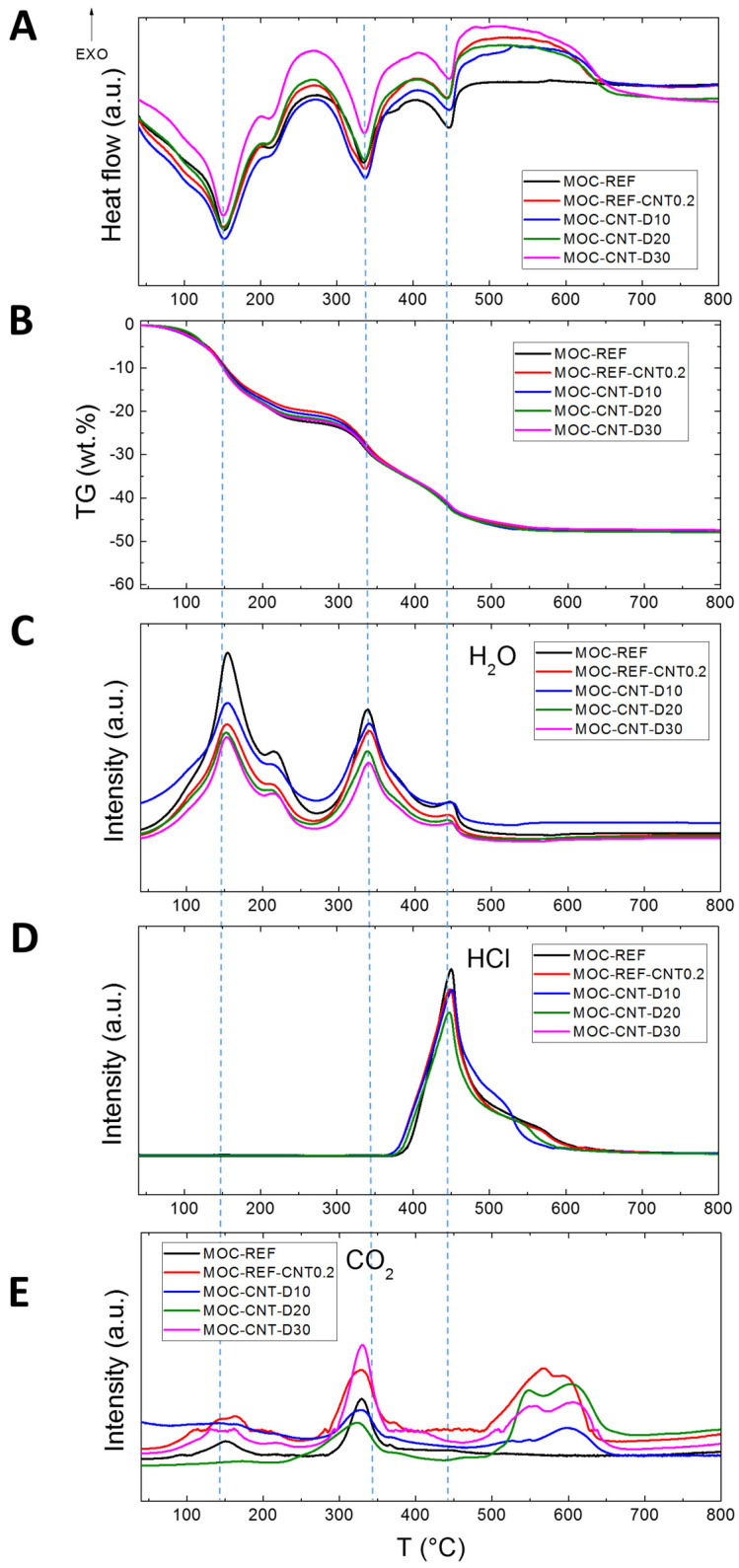
STA-MS of prepared composites: (**A**) DTA, (**B**)TG, (**C**) MS of water, (**D**) MS of hydrochloric acid and (**E**) MS for carbon dioxide. The blue dashed line is showing the position of three major endothermic effects.

The STA-MS was used to study the thermal stability of the samples (see Figure 5). All samples gradually decomposed, as can be observed from DTA and TG curves and also from MS signal for water, carbon dioxide and hydrochloric acid. After the heating, only magnesium oxide remained, while other products are gases. The DTA curve shows three major endothermic effects (marked by a blue dashed line). The first one at approximately 150 °C is associated with water release. Moreover, the second effect at approximately 340 °C is caused by water release, while the third effect at 445 °C is connected mainly due to the release of hydrochloric acid. The major difference between REF and other samples with MWCNT can be observed in the area between 450 and 650 °C, where a wide exothermic effect was detected. This is due to the oxidation of MWCNT, which was also confirmed by the MS spectrum for carbon dioxide (Figure 5E). Let us note that carbon dioxide was also released between 300 and 350 °C. This was probably caused by the decomposition of chlorartinite Mg_2_(CO_3_)(OH)Cl·3H_2_O, which usually forms on the surface of the samples due to CO_2_ capture.

**Figure 6:**
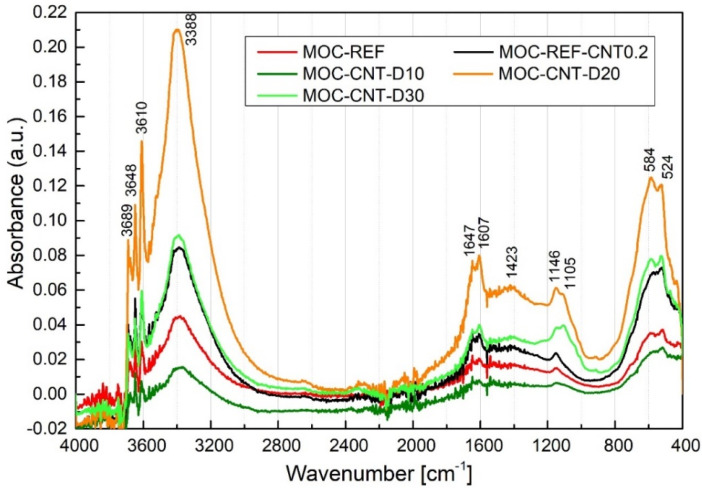
The MIR spectrum of tested composites in the range of 400–4000 cm^−1.^

The collected MIR (medium infrared) spectra are presented in the form of spectral lines in Figure 6. The major absorption bands are precisely assigned and summarised in the Supplementary Materials. As we can observe, all lines are very similar and differ only in detail. The main differences can be observed in absorption bands around 1600 cm^−1^. The stretching vibration of H–O–H in water in minerals around 1100 cm^−1^, where the intensive band at 1050 cm^−1^ coming from the in-plane vibration of Si–O–Si in quartz in MOC-REF and MOC-REF-CNT0.2, is broken into the bands between 1050 cm^−1^ and 1105 cm^−1^ in MOC-CNT-D composites [13]. This can be assigned to the asymmetric in-plane stretching vibration of Si–O–Si in diatomite and Si–OH in calcium silicates and magnesium silicates. From the comparison of reference magnesium oxychloride cement sample and magnesium oxychloride cement with nanotubes, the presence of C=C bond is demonstrated at 1560 cm^−1^. This vibration is moved to higher wavenumbers 1564 cm^−1^ in the case of composites with diatomite. The reflection of Si–O, Si–O–Al or Si–O–Si bonds coming from diatomite can be observed as stretching vibration in the range below 800 cm^−1^. In the range between 3300 and 3700 cm^−1^, the symmetric and asymmetric stretching mode of O–H bonds can be observed. In order to explore material behaviour, the most important is the band at 3689 cm^−1^, which can be attributed to the stretching vibration of OH group in brucite in H_2_O and Mg(OH)_2_ [4]. The intensities decreased nearly half in the case of samples with 20 and 30 wt.% of diatomite, which means the poor formation of brucite that results in higher mechanical properties. Similarly, the formation of silicate hydrates influences the mechanical properties of prepared composites. The most intensive band can be observed in the case of the sample with 10 wt.% of diatomite, while the others show lower content of silicate hydrates.

The spectral range around 2000 cm^−1^ was not analysed in detail, the low intensive bands were made up of bending and rocking vibrations of O–H in water and brucite and carbonates overtones. The band intensities between 1715 cm^−1^ and 1150 cm^−1^ are stretching and bending vibrations of Mg–O and O–H bonds in MgCl_2_·8H_2_O and C-S-H or M-S-H phases, respectively [3,4]. The presence of MgCO_3_ in light burned magnesia and from the carbonation reaction can be observed as the stretching vibration of C=O in carbonates at 1488 cm^−1^ [11]. The series of modes below 1000 cm^−1^ originated from the lattice translation and bond vibrations Mg–O/Mg–Cl and vibrations of Mg–O/Mg^2+^, O/O–Mg–O/O–Mg^2+^–O bonds, rocking motion of oxygen atoms bridging silicon and aluminium atoms in bonds Si–O–Si and Si–O–AlIV [14].

The macrostructural parameters of the composites after 28 days are presented in Table 3. The presented data represent mean values measured independently for five samples. The expanded combined uncertainty of the obtained parameters is also introduced. It is evident that the use of MWCNTs reduced the porosity of the prepared composites. The partial replacement of MgO, which was used as finer filler, by fine-grained diatomite also led to the reduction in total open porosity. However, in this case, the drop in porosity was much lower than the drop caused by MWCNTs solely.

**Figure 7:**
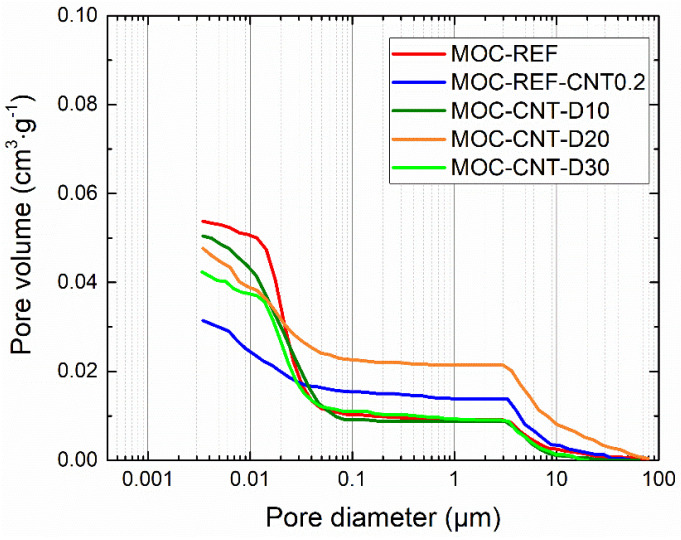
Cumulative intruded pore volume.

**Figure 8:**
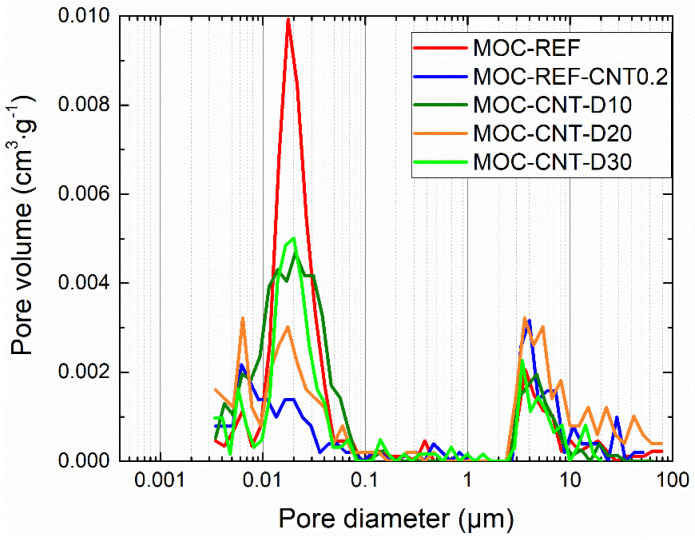
Incremental intruded pore volume.

The cumulative and pore size distribution curves obtained by MIP (mercury intrusion porosimetry) are presented in Figure 7 and Figure 8. The microstructural data are presented in Table 4. Classification of pores originally applied in Portland cement-based materials research was adopted in literature [15]. In the analyzed composite, three classes of pores were identified: i) gel pores (<10 nm), which govern shrinkage and creep of the matrix components; ii) capillary pores (10–10,000 nm) driving the mechanical strength and transport processes; and iii) hollow shell pores (>0.1 mm) for which its presence negatively affects the mechanical resistance. The use of MWCNTs and diatomite resulted in a substantial decrease in pore volume in the 0.01–0.1 µm diameter range, which well corresponds with the results of the mechanical and hygric properties that were, thus, greatly improved by the reduction in capillary pores volume. The total porosity data measured by MIP analysis were almost similar to the data calculated from the bulk density and specific density values. Both the total cumulative volume and average pore diameter were reduced by the addition of MWCNTs and admixing of diatomite. This evinces the condensation and refinement of the MOC matrix, which was visible for all diatomite and MWCNTs doped composites.

**Figure 9:**
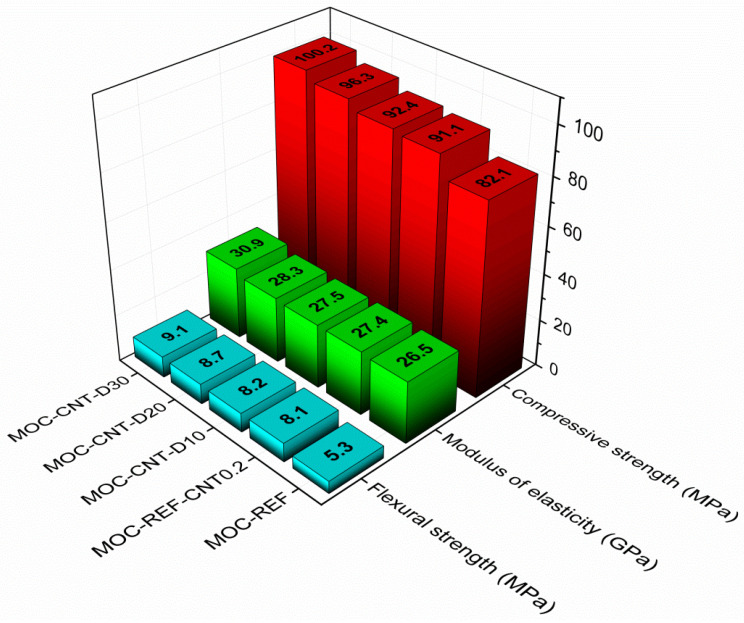
Mechanical parameters of 28 days composites.

The mechanical parameters of the investigated composites measured for 28 days hardened samples are graphically presented in Figure 9. Diatomite, as well as MWCNTs, greatly enhanced the mechanical strength and stiffness of the analyzed materials, and their synergic performance was, thus, well proven. Generally, all tested composites yielded excellent mechanical parameters, which were further improved by a coupled diatomite and MWCNTs effect. High compressive strengths together with high modulus of elasticity are typical properties of MOC-based materials and enable inmixing of different types of aggregates and fillers into the composite mixture without the immoderate loss of the mechanical parameters of the hardened composites. Moreover, with the higher replacement ratio of MgO with diatomite, the investigated mechanical parameters increased. This is a very promising result, especially due to the low carbon footprint of diatomite production compared to the expansive and polluting manufacturing of MgO. In this manner, high-strength composites can be obtained by more efficient and eco-friendly methods. Quantitatively, in the comparison with reference material MOC-REF, the increments in the compressive strength were 11.0%, 12.5%, 17.3% and 22.0 for composites MOC-REF-CNT0.2, MOC-CNT-D10, MOC-CNT-D20 and MOC-CNT-D30, respectively. A similar trend was also exhibited by the other two tested mechanical parameters, which were increased by 52.8%, 54.7%, 64.2% and 71.7% in the case of flexural strength and 3.4%, 3.8%, 6.8% and 16.6% for the modulus of elasticity values. The improvement in the stiffness of the composites was the lowest among the examined mechanical parameters, but still significant.

The mechanical parameters correspond well with the macrostructural and microstructural parameters data. The decrease in the volume of capillary pores is the reason for the improvement of mechanical resistance and stiffness of the composites with MWCNTs themselves or combined with diatomite of high Blaine fineness. The contribution of diatomite to the total mechanical resistance is due to two phenomena: (i) The filler effect of diatomite filling the gaps between the MgO particles and precipitated MOC-based compounds; (ii) the reactivity of diatomite and formation of magnesium silicate hydrate (M-S-H) phases. Thermal parameters of the studied samples are introduced in Table 5. These are the results of the mutual combination of several effects: (i) porosity and, thus, bulk density, (ii) low thermal conductivity and specific heat of diatomite frustules [16,17] and (iii) high thermal conductivity and specific heat of CNTs [18,19]. In MOC-REF-CNT0.2, the effect of CNTs addition prevailed, whereas the use of CNTs increased both the thermal conductivity and specific heat. Similar enhancement of heat transport by the incorporation of CNTs was reported by Hassanzadeh-Aghdam et al. [20] who introduced 1 vol.% of CNTs in concrete, and the thermal conductivity was observed to increase by 11.1%. In our case, due to the lower dosage of CNTs in the composite mixtures, the increase in the thermal conductivity was 3.6%. In MOC-CNT-D composites, the low thermal conductivity of diatomite produced reduced thermal conductivity values, and the drop in thermal conductivity was partially compensated by the decreased porosity of these materials in comparison to that of MOC-REF. On the contrary, the higher bulk density of MOC-CNT-D materials slightly enhanced the heat storage capability, which can find application, e.g.*,* in passive moderation of indoor air climate. In case of the higher dosage of CNTs and their optimum disperison, good networking of CNTs can be anticipated and, thus, more significantly enhanced heat transport.

The parameters characterizing water ingress into the researched composites are presented in Table 6. The addition of MWCNTs into the composites reduced the water transport, which is positive due to the partial elimination of the risk of moisture-induced damage. The partial MgO replacement with diatomite slightly devaluated the water transport retardation effect of CNTs due to the highly porous microstructure of diatomite skeletons, but MOC-CNT-D materials still yielded lower water transport parameters than the parameters obtained for MOC-REF. In this sense, the low porosity of magnesium silicate hydrates can partially reduce the total water ingress and rate of moisture transport. Quantitatively, the water absorption coefficient was low for all tested samples, which is favorable for the durability of the composites with respect to their deterioration in the presence of water and reactive MgO. Capillary active materials have the water absorption coefficient typically about one order of magnitude higher [21,22,23] than measured for MOC-based derivatives examined in this work.

## 4. Conclusions

In this study, the combined effect of two additives, MWCNTs and diatomite, on MOC-based construction materials was studied. The addition of such dopants resulted in the following properties of the final material:

1. The use of very low content of MWCNT (0.2 wt.%) caused a large decrease in the total open porosity: For the sample MOC-REF-CNT0.2, the decrease was approximately 36% in comparison with the reference sample. The decrease was caused by the good dispersion of the nanoadditive, which resulted in filling the capillary pores of the matrix. This property also manifested in the samples containing diatomite; however, the decrease was lower due to the content of the secondary filler.
2. The use of both additives caused an improvement in the mechanical parameters, namely the compressive strength, flexural strength and the modulus of elasticity. The biggest increase was measured for the sample containing 30 wt.% of diatomite as a partial replacement for the MgO filler, where the value of the compressive strength reached 100.2 MPa, which is about 22 % higher than the value obtained for the reference sample.
3. The positive influence of the additives was also manifested in the decrease in the water transport abilities, which is a very crucial parameter for the MOC-based materials. The increase in water-resistance of such materials is critical for their possible application.

Overall, these results represent a very promising route for future research and application of the MOC-based composite materials as well as the use of carbon-based nanoadditives and secondary fillers, such as diatomite, in the building industry.

## Figures and Tables

**Table 1:** Mixture properties of prepared samples.

Mixture ID	Mass (g)				
	MgO	MgCl_2_·6H_2_O	H_2_O	Diatomite	MWCNT
MOC-REF	553.6	399.0	247.5	-	-
MOC-REF-CNT0.2	552.5	398.2	247.0	-	2.4
MOC-CNT-D10	536.7	398.2	247.0	15.8	2.4
MOC-CNT-D20	521.0	398.2	247.0	31.6	2.4
MOC-CNT-D30	505.2	398.2	247.0	47.4	2.4

**Table 2:** Basic physical characteristics of diatomite and MgO.

Substance	Specific Density (kg·m^−3^)	Powder Density (kg·m^−3^)	Blaine Fineness (m^2^·kg^−1^)	Particle Size		
				d_10_	d_50_	d_90_
Diatomite	2416	302	2095	5.5	19.9	44.0
MgO	341	837	693	0.8	2.47	8.1

**Table 3:** Macrostructural parameters of 28 days matured composites.

Composite	Bulk Density *ρ_b_* (kg∙m^−3^)	Specific Density *ρ_s_* (kg∙m^−3^)	Total Open Porosity *Ψ* (%)
MOC-REF	1779 ± 25	1957 ± 23	9.1 ± 0.2
MOC-REF-CNT-0.2	1808 ± 25	1918 ± 23	5.8 ± 0.1
MOC-CNT-D10	1760 ± 25	1924 ± 23	8.5 ± 0.2
MOC-CNT-D20	1756 ± 25	1912 ± 23	8.2 ± 0.2
MOC-CNT-D30	1741 ± 24	1890 ± 23	7.9 ± 0.2

**Table 4:** Microstructural parameters of the hardened composites determined by MIP.

Composite	Hg Porosity (%)	Total Pore Volume (cm^3^·g^−1^)	Average Pore Diameter (µm)
MOC-REF	9.5	0.0538	0.0208
MOC-REF-CNT0.2	5.6	0.0314	0.0071
MOC-CNT-D10	8.8	0.0506	0.0199
MOC-CNT-D20	8.4	0.0476	0.0176
MOC-CNT-D30	8.0	0.0425	0.0142

**Table 5:** Thermal properties of 28 days matured composites.

Composite	*λ* (W·m^−1^·K^−1^)	*c_v_* × 10^6^ (J·m^−3^·K^−1^)
MOC-REF	1.107	2.128
MOC-REF-CNT0.2	1.147	2.404
MOC-CNT-D10	1.007	2.215
MOC-CNT-D20	1.022	2.235
MOC-CNT-D30	1.041	2.275

**Table 6:** Hygric properties of 28 days matured composites.

Composite	*A*_w_ (kg·m^−2^·s^−1/2^)	*W_a_* × 10^6^ (J·m^−3^·K^−1^)
MOC-REF	0.038	3.941
MOC-REF-CNT0.2	0.022	2.350
MOC-CNT-D10	0.034	3.512
MOC-CNT-D20	0.030	3.356
MOC-CNT-D30	0.026	3.094

## Data Availability

The data presented in this study are available upon request from the corresponding author. The data are not publicly available due to privacy reasons.

## Supplementary Materials

The following are available online at https://www.mdpi.com/article/10.3390/ma14164576/s1, Figure S1: The TEM micrographs of the used multi-walled carbon nanotubes, Figure S2: The micrographs of the diatomite powder obtained from SEM, Figure S3: Arrangement of samples and placing of sensor in Hot Disk measurement, Table S1: Assignments of the major absorption bands of MOC composites.

## References

[1] Canterford J.H. (1985). Magnesia-an important industrial mineral: A review of processing options and uses. Miner. Process. Extr. Matell. Rev..

[2] Bilinski H., Matkovicć B., Mažuranicć C., Žunicć T. (1984). The formation of magnesium oxychloride phases in the systems MgOMgCl2-H2O and NaOH-MgCl_2_-H_2_O. J. Am. Ceram. Soc..

[3] Lojka M., Jankovský O., Jiříčková A., Lauermanova A.M., Antončík F., Sedmidubský D., Pavlík Z., Pavlíková M. (2020). Thermal Stability and Konetics of Formation of Magnesium Oxychloride Phase 3Mg(OH)(2). MgCl2.8H(2)O. Materials.

[4] Jiříčková A., Lojka M., Lauermanova A.M., Antončík F., Sedmidubský D., Pavlíková M., Záleská M., Pavlík Z., Jankovský O. (2020). Synthesis, Structure, and Thermal Stability of Magnesium Oxychloride 5Mg(OH)_2_·MgCl_2_·8H_2_O. Appl. Sci..

[5] Hao Y., Li Y. (2021). Study on preparation and properties of modified magnesium oxychloride cement foam concrete. Constr. Build. Mater..

[6] Zhou X., Li Z. (2012). Light-weight wood–magnesium oxychloride cement composite building products made by extrusion. Constr. Build. Mater..

[7] Gomes C.M., Cheung N., Gomes G.M., Sousa A.K., Peruzzi A.P. (2021). Improvement of water resistance in magnesia cements with renewable source silica. Constr. Build. Mater..

[8] Bensted J., Hewlett P.C. (2003). Special cements. Lea’s Chemistry of Cement and Concrete.

[9] Lauermanova A.M., Lojka M., Jankovský O., Faltysová I., Pavlíková M., Pivák A., Záleská M., Pavlík Z. (2021). High-performance magnesium oxychloride composites with silica sand and diatomite. J. Mater. Res. Technol..

[10] Yılmaz B., Ediz N. (2008). The use of raw and calcined diatomite in cement production. Cement Concr Compos.

[11] Guo Y., Zhang Y., Soe K., Hutchinson W.D., Timmers H., Poblete M.R. (2020). Effect of fly ash on mechanical properties of magnesium cement under water attack. Struct. Conc..

[12] Zhang T., Vandeperre L.J., Cheeseman C.R. (2014). Formation of magnesium silicate hydrate (M-S-H) cement pastes using sodium hexametaphosphate. Cem. Concr. Res..

[13] Bernard E., Lothenbach B., Cau-Dit-Coumes C., Chlique C., Dauzères A., Pochard I. (2018). Magnesium and calcium silicate hydrates, Part I: Investigation of the possible magnesium incorporation in calcium silicate hydrate (C-S-H) and of the calcium in magnesium silicate hydrate (M-S-H). Appl. Geochem..

[14] Sugimoto K., Robert E., Dinnebiera R.E., Hansonb J.C. (2007). Structures of three dehydration products of bischofite from in situ synchrotron powder diffraction data (MgCl2nH2O; n = 1, 2, 4). Acta Cryst..

[15] Zhao H., Xiao Q., Huang D., Zhang S. (2014). Influence of Pore Structure on Compressive Strength of Cement Mortar. Sci. World J..

[16] Benayache S., Alleg S., Mebrek A., Suñol J.J. (2018). Thermal and microstructural properties of paraffin/diatomite composite. Vacuum.

[17] Jeong S.-G., Jeon J., Lee J.-H., Kim S. (2013). Optimal preparation of PCM/diatomite composites for enhancing thermal properties. Int. J. Heat Mass Transf..

[18] Zhao Y.H. (2014). Effect of CNT/CNF on Thermal and Mechanical Properties of Cement Mortars. Adv. Mater. Res..

[19] Zhu Y., Qian Y., Zhang L., Bai B., Wang X., Li J., Bi S., Kong L., Liu W., Zhang L. (2021). Enhanced thermal conductivity of geopolymer nanocomposites by incorporating interface engineered carbon nanotubes. Compos. Commun..

[20] Hassanzadeh-Aghdam M.K., Mahmoodi M.J., Safi M. (2019). Effect of adding carbon nanotubes on the thermal conductivity of steel fiber-reinforced concrete. Compos. Part B: Eng..

[21] Feng C., Guimarães A.S., Ramos N., Sun L., Gawin D., Konca P., Hall C., Zhao J., Hirsch H., Grunewald J. (2020). Hygric properties of porous building materials (VI): A round robin campaign. Build. Environ..

[22] Veiga M.R., Magalhães A., Bosilijkov V., Martens D., Vermeltfoort A. (2004). Capillarity tests on historic mortar samples extracted from site. Methodology and Compared Results, Proceedings of the 13th International Brick and Block Masonry Conference, Amsterdam, The Netherlands, 4–7 July 2004.

[23] Vyšvařil M., Pavlíková M., Záleská M., Pivák A., Žižlavský T., Rovnaníková P., Bayer P., Pavlík Z. (2020). Non-hydrophobized perlite renders for repair and thermal insulation purposes: Influence of different binders on their properties and durability. Constr. Build. Mater..

