# Supplementary material for: MOC-Diatomite Composites Filled with Multi-Walled Carbon Nanotubes
Source: Materials (Basel). 2021 Aug 15;14(16):4576. doi: 10.3390/ma14164576 (PMC8399920; doi:10.3390/ma14164576)
Supplement: Supplementary file 1 [file materials-14-04576-s001.zip › materials-1318010-supplementary.pdf]

materials

MDPI

Supplementary Material

MOC-Diatomite Composites Filled with Multi-Walled Carbon Nanotubes

Milena Pavlíková[1,*], Martina Záleská[1], Adam Pivák[1], Ondřej Jankovský[2], Anna-Marie Lauermannová[2], Michal Lojka[2], Filip Antončík[2] and Zbyšek Pavlík[1]

[1] Department of Materials Engineering and Chemistry, Faculty of Civil Engineering, Czech Technical University in Prague, Thákurova 7, 166 29 Prague 6, Czech Republic; martina.zaleska@fsv.cvut.cz (M.Z.); adam.pivak@fsv.cvut.cz (A.P.); pavlikz@fsv.cvut.cz (Z.P.)

[2] Department of Inorganic Chemistry, Faculty of Chemical Technology, University of Chemistry and Technology, Technická 5, 166 28 Prague 6, Czech Republic; ondrej.jankovsky@vscht.cz (O.J.); Anna-marie.Lauermannova@vscht.cz (A.-M.L.); michal.lojka@vscht.cz (M.L.); Filip.Antoncik@vscht.cz (F.A.)

* Correspondence: milena.pavlikova@fsv.cvut.cz; Tel.: +420 224 354 371

Citation: Pavlíková, M.; Záleská, M.; Pivák, A.; Jankovský, O.; Lauermannová, A.-M.; Lojka, M.; Antončík, F.; Pavlík, Z. MOC-Diatomite Composites Filled with Multi-Walled Carbon Nanotubes. *Materials* **2021**, *14*, 4576. https://doi.org/10.3390/ma14164576

Academic Editor: Hyeong-Ki Kim

Received: 13 July 2021
Accepted: 13 August 2021
Published: 15 August 2021

Publisher's Note: MDPI stays neutral with regard to jurisdictional claims in published maps and institutional affiliations.

Copyright: © 2021 by the authors. Submitted for possible open access publication under the terms and conditions of the Creative Commons Attribution (CC BY) license (http://creativecommons.org/licenses/by/4.0/).

Materials and Methods

The materials used in this work for binder preparation were light magnesium oxide (MgO) possessing the declared purity of > 98 %, obtained from Penta, s.r.o., Czech Republic, and magnesium chloride hexahydrate ($MgCl_2{\cdot}6H_2O$) of p.a. purity from Lach-Ner, s.r.o., Czech Republic. The multi-walled carbon nanotubes (MWCNTs and TNIM8) of purity > 95 % were purchased from TimesNano, China. Their microstructure, which was obtained from TEM, is pictured in Figure S1. Diatomite (SEM micrographs are shown in Figure S2) was produced by LB Minerals, s.r.o., Czech Republic. The chemical composition of the diatomite powder was determined by X-ray fluorescence analysis (ARL QUANT'X EDXRF Spectrometer, Thermo Scientific, City, State abbreviation, USA) and resulted in 81.7 % SiO_2, 13.8 % Al_2O_3, 1.6 % Fe_2O_3 and traces of K_2O and MgO.

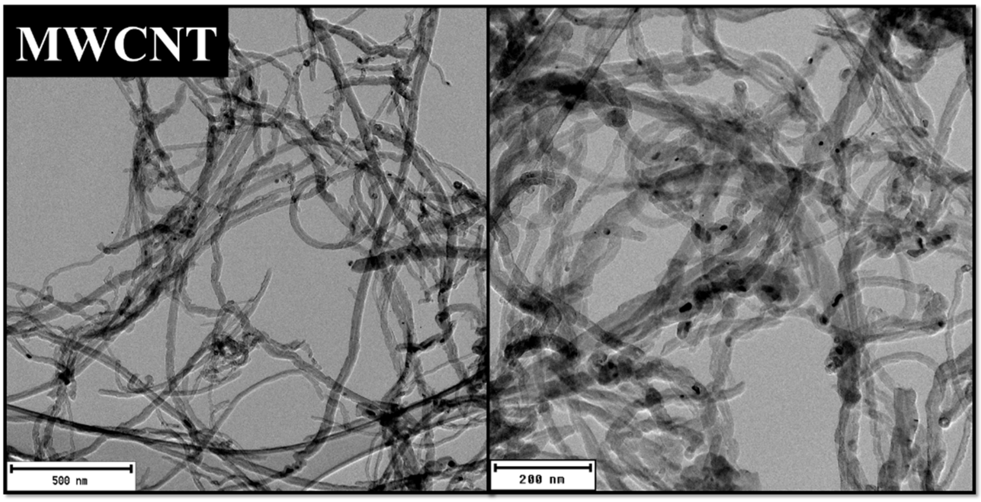

Figure S1. The TEM micrographs of the used multi-walled carbon nanotubes.

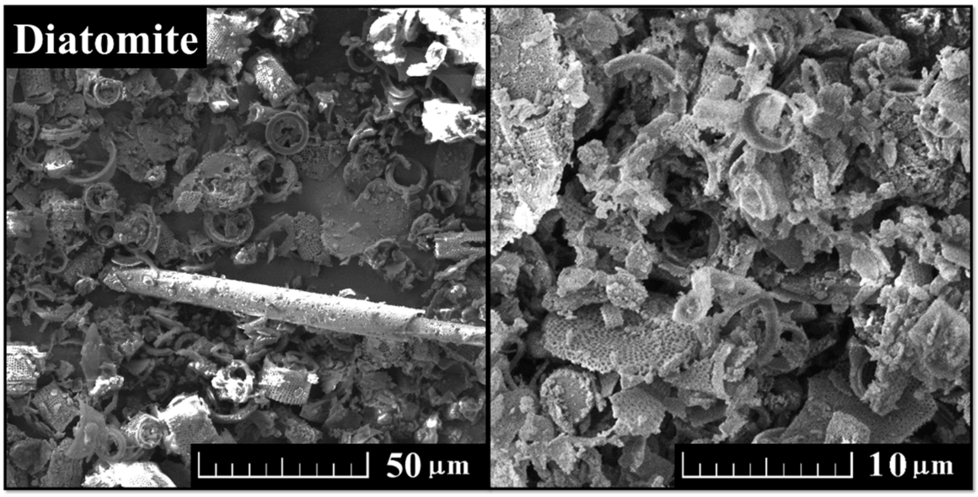

Figure S2. The micrographs of the diatomite powder obtained from SEM**.**

The specific density was measured by means of helium pycnometry. The powder density was studied by the measurement of sample mass in a graduated cylinder. The specific surface was tested by the Blaine air-permeability apparatus using the procedure specified in EN 196-6 [1]. Laser particle size analyzer Analysette 22 MicroTec plus (Manufacture, Fritsch, Germany) was used for the analysis of particle size distribution.

Optical microscopy of the composite samples was performed by using a Navitar macro-optics (Manufacture, Rochester, State abbreviation, USA) microscope with optical zoom up to 110× and recorded with a digital camera Sony 2/3" with a resolution of 5 Mpix. The sample was illuminated by a white LED ring light source with individually addressable segments and intensity. NIS-Elements BR 5.21.02 software (Version, Manufacture, City, State abbreviation, Country) with an Extended Depth of Focus Module (EDF) was used for imagining and analysis of the samples.

XRD analysis was carried out by Bruker D2 Phaser (Bruker, Karlsruhe, Germany), and powder diffractometry was employed with Bragg–Brentano geometry, applying CuKα radiation (λ = 0.15418 nm, U = 30 kV, I = 10 mA) and sample rotation (5 rpm). The step size was set to 0.02025° (2θ), and the overall data were collected from the angular range of 5°–80°.

In order to study the surface morphology of the samples, SEM performed on a Tescan MAIA 3 (Tescan Brno, Brno, Czech Republic) was used. The elemental composition and maps were obtained by using an energy dispersive spectroscopy (EDS) analyzer (X-Max150) with a 20 mm^2 SDD detector (Oxford Instruments, Oxfordshire, UK) and AZtecEnergy software. The sample was held on carbon conductive tape in order to ensure the conductivity of the experiments. For both SEM and SEM-EDS analysis, the electron beam was set to 10 kV, with a 10 mm work distance.

HR-TEM was performed using an EFTEM Jeol 2200 FS microscope (Jeol, Tokyo, Japan). A 200 keV acceleration voltage was used for the measurement. The sample preparation was attained by drop-casting the suspension (1 mg mL^{-1} in isopropyl alcohol) on a TEM grid (Cu; 200 mesh; Formvar/carbon) and then drying in a vacuum dryer at 25 °C and $p/p_0 = 0.2$.

In order to investigate the influence of different diatomite dosage on composites formation, the MIR spectra using ATR technique were collected. For the comparison, reference samples only composed of magnesium oxychloride cement and a sample of magnesium oxychloride cement with 0.2 wt.% of nanotubes were also tested. The FT-IR spectrometer Nicolet 6700 (Thermo Fisher Scientific, Waltham, MA, USA) worked in wavenumbers range from 4000 to 400 cm^{-1}, with spectral resolution 4 cm^{-1}. Each spectrum was finalized after 32 scans. The analyzed samples were firstly dried in an oven at 105 °C to

remove physically bonded water. Then, they were crushed and consequently homogenized with ball grinder MM 400 (RETSCH) (Manufacture, City, State abbreviation, Country).

Simultaneous thermal analysis (STA) was performed by the Setsys Evolution apparatus from Setaram with a temperature up to 800 °C. The measurements were performed in a dynamic air atmosphere with the flow rate 50 mL.min^{-1} and heating rate to 5 °C·min^{-1}. The mass spectrometer OmniStar™ from Pffeifer Vacuum was used in order to analyze gases that evolved during the heating.

The hardened composites were tested in the age of 28 days. The tested fundamental macrostructural parameters were dry bulk density ρ_b (kg·m^{-3}), specific density ρ_s (kg·m^{-3}) and total open porosity ψ (-). For each composite type, 5 samples were tested. The dry bulk density was measured in accordance with the standard EN 1015-10 [2]. Helium pycnometer Pycnomatic ATC (Porotec, Germany) was used for the specific density assessment. From the determined bulk density and specific density values, the total open porosity was simply calculated [3]. The expanded combined uncertainties of the investigated macrostructural parameters were 1.4 %, 1.2 % and 2.0 % for the dry bulk density, specific density and total open porosity, respectively.

The microstructure of the prepared composites was studied by mercury porosimeters of Pascal series, Pascal 140 and Pascal 440 (Thermo Fisher Scientific, City, State abbreviation, Country)). The circular cross-section of capillaries was assumed in the evaluation of the mercury intrusion porosimetry (MIP) data. In the MIP test, dry samples with a mass of about 1.5 g were analyzed.

Flexural strength f_f (MPa) and compressive strength f_c (MPa) were measured in compliance with EN 1015-11 [4]. For each material, 5 samples were examined in both strength tests. The specimens were cast as 40 mm × 40 mm × 160 mm prisms, and the rest of the broken prisms was used for the compressive strength measurement, i.e., the uniaxial loading force was applied on 40 mm × 40 mm cross-section of samples. As prescribed in the above-referred standard, the applied load speed was 50 N·s^{-1} in the flexural strength testing and 100 N·s^{-1} in the compressive strength assessment. Another investigated mechanical parameter was the dynamic modulus of elasticity E_d (GPa), which was tested by the Vikasonic apparatus (Schleinbinger Geräte, Buchbach, Germany) operating on a frequency of 54 kHz. The measurement was conducted in the longitudinal axes of casted prisms. The expanded combined uncertainty of the mechanical parameter assessment was 1.4 %, 1.4 % and 2.3 % for f_f, f_c and E_d, respectively.

Water-induced damage is the biggest disadvantage of MOC-based materials and products. Therefore, the effect of CNTs and diatomite on water transport parameters of the developed composites was researched. The investigated parameters were water absorption coefficient A_w (kg·m^{-2}·s$^{-1/2}$) and 24 h water absorption W_a (kg·m^{-2}). These were obtained in accordance with the EN 1015-18 [5]. The uncertainty in the assessment of hygric parameters was 2.3 % for A_w and 1.2 % for W_a.

It was anticipated that the use of CNTs and diatomite with its highly porous structure will affect the heat transport and storage in a significant manner. Therefore, thermal conductivity λ (W·m^{-1}·K^{-1}) and volumetric heat capacity c_v (J·m^{-3}·K^{-1}) of the hardened samples were tested by using a transient plane source technique [6,7] working device Hot Disk TPS 1500 (Hot Disk AB, Göteborg, Sweden). For the measurement, the caption sensor (radius of 6.4 mm) originally provided by the producer was placed on the surface of the dry sample, which was placed in the measurement chamber. The temperature of the test was 23 ± 2 °C. The caption sensor located between the tested specimens in a measuring unit is shown in Figure S3.

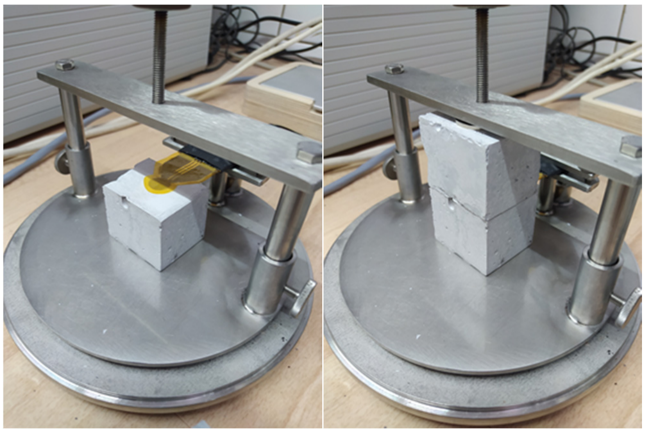

Figure S3. Arrangement of samples and placing of sensor in Hot Disk measurement.

Results

The major absorption bands obtained from MIR are precisely assigned and summarised in the Table S1.

Table S1. Assignments of the major absorption bands of MOC composites.

Wavenumbers (cm^{-1})	Assignment
3689	stretching (ν) vibration of O-H in $Mg(OH)_2$
3677	stretching (ν) vibration of O-H in crystalline hydroxyl
3648, 3565	stretching (ν) vibration of O-H in silicate hydrates
3586, 3612-3608, 3526	stretching (ν) vibration of H-O-H in $MgCl_2{\cdot}8H_2O$
3388	stretching (ν) vibration of H-O-H in H_2O
2050	bending (δ) and rocking ( ) of H-O-H in H_2O
1715, 1704, 1681, 1621, 1423	stretching (ν) vibration of Mg-O in $MgCl_2{\cdot}8H_2O$
1647, 1607, 1150	bending (δ) vibration of H-O-H in $MgCl_2{\cdot}8H_2O$
1560	stretching (ν) vibration of C=C
1541, 1521	scissoring vibration of H-O-H
1488	stretching (ν) C=O in $MgCO_3$
1455-1423	stretching (ν) O-H in C-S-H and M-S-H phase,
1146	bending (δ) vibration of H-O-H in $MgCl_2{\cdot}8H_2O$
592-584	deformation (δ) and stretching (ν) lattice vibrations of Mg-Cl/Mg-O
517-524, 457	translation vibrations of Mg/Mg-O, Mg-OH, and stretching (ν) vibration of O-Si-O in diatomite
429	bending (δ) vibration of O-Si-O in diatomite
414	vibrational modes of the lattice showing the Mg-O/Mg^{2+}, O/O-Mg-O/O-Mg^{2+}-O bonds

References

1. EN 196-6, Methods of testing cement - Part 6: Determination of fineness. European Committee for Standardization: Brussels, Belgium, 2018.
2. EN 1015-10, Methods of Test for Mortar for Masonry—Part 10: Determination of Dry Bulk Density of Hardened 676 Mortar. European Committee for Standardization: Brussels, Belgium, 1999.
3. Záleská, M.; Pavlík, Z.; Čítek, D.; Jankovský, O.; Pavlíková, M. Eco-friendly concrete with scrap-tyre-rubber-based aggregate – Properties and thermal stability. *Construction and Building Materials* **2019**, *225*, 709-722, doi:https://doi.org/10.1016/j.conbuildmat.2019.07.168.
4. EN 1015-11, Methods of Test for Mortar for Masonry-Part 10: Determination of Flexural and Compressive Strength 678 of Hardened Mortar. European Committee for Standardization: Brussels, Belgium, 1999.

5. EN 1015-18: Methods of Test for Mortar for Masonry—Part 18: Determination of Water Absorption Coefficient Due to Capillary Action of Hardened Mortar. European Committee for Standardization: Brussels, Belgium, 2002.
6. Gustafsson, S.E. Transient plane source techniques for thermal conductivity and thermal diffusivity measurements of solid materials. *Review of Scientific Instruments* **1991**, *62*, 797-804, doi:10.1063/1.1142087.
7. Pavlík, Z.; Jerman, M.; Trník, A.; Kočí, V.; Černý, R. Effective thermal conductivity of hollow bricks with cavities filled by air and expanded polystyrene. *Journal of Building Physics* **2013**, *37*, 436-448, doi:10.1177/1744259113499214.
